# Left ventricular hypertrophy diagnosed after a stroke: a case report

**DOI:** 10.1186/s13256-018-1592-4

**Published:** 2018-03-22

**Authors:** Wilfred Ifeanyi Umeojiako, Ritesh Kanyal

**Affiliations:** Medway Maritime Hospital, Windmill road, Gillingham, ME7 5NY UK

**Keywords:** Stroke, Electrocardiogram, Left ventricular hypertrophy

## Abstract

**Background:**

Stroke is a recognized clinical course of hypertrophic cardiomyopathy. This interesting case showed notable difference on the electrocardiogram of a patient 4 months prior to suffering a stroke and 10 days after suffering a stroke. The pre-stroke electrocardiogram showed atrial fibrillation with a narrow QRS complex, while the post-stroke electrocardiogram showed marked left ventricular hypertrophy. Left ventricular hypertrophy was diagnosed using the Sokolow-Lyon indices. The development of left ventricular hypertrophy a few days after suffering a stroke has not previously been reported.

**Case presentation:**

An 83-year-old white British woman with a background history of permanent atrial fibrillation, hypertension, and previous stroke attended the emergency department with a 2-day history of exertional dyspnea, and chest tightness. On examination, she had bibasal crepitations with a systolic murmur loudest at the apex.

In-patient investigations include an electrocardiogram, blood tests, chest X-ray, contrast echocardiogram, coronary angiogram, and cardiovascular magnetic resonance imaging. An electrocardiogram showed atrial fibrillation, with inferolateral T wave inversion, and left ventricular hypertrophy. A chest X-ray showed features consistent with pulmonary edema. A contrast echocardiogram showed marked hypertrophy of the mid to apical left ventricle, appearance consistent with apical hypertrophic cardiomyopathy. Coronary angiography showed eccentric shelf-type plaque with non-flow-limiting stenosis in the left coronary artery main stem. Cardiovascular magnetic resonance imaging reported findings highly suggestive of apical hypertrophic cardiomyopathy. Our patient was treated and discharged on rivaroxaban, bisoprolol, and atorvastatin with a follow-up in the cardiomyopathy outpatient clinic.

**Conclusions:**

Electrocardiogram diagnosis of left ventricular hypertrophy led to the diagnosis of apical hypertrophic cardiomyopathy in this patient. Left ventricular hypertrophy was only evident a few days after our patient suffered a stroke. The underlying mechanisms responsible for this remain unclear. Furthermore, differential diagnosis of hypertrophic cardiomyopathy should be considered in people with electrocardiogram criteria for left ventricular hypertrophy. Cardiovascular magnetic resonance imaging is an important diagnostic tool in identifying causes of left ventricular hypertrophy. Family screening should be recommended in patients with new diagnosis of hypertrophic cardiomyopathy.

## Background

While stroke is a recognized clinical course of hypertrophic cardiomyopathy (HCM), it is not known to unmask left ventricular hypertrophy (LVH) on electrocardiogram (ECG). It is widely accepted that LVH can develop due to intrinsic stimuli such as cardiomyopathy, or extrinsic stimuli such as hypertension [1]. It is also important to note that LVH is not progressive in the majority of patients [1].

**Fig. 1:**
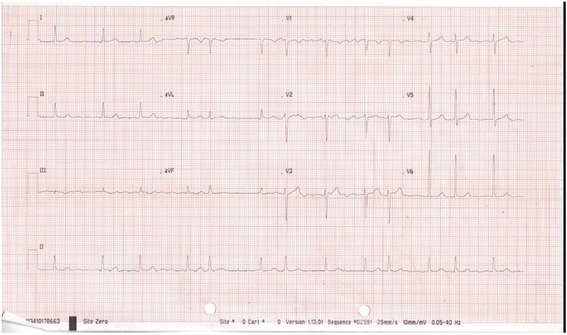
Patient’s electrocardiogram before suffering a stroke

**Fig. 2:**
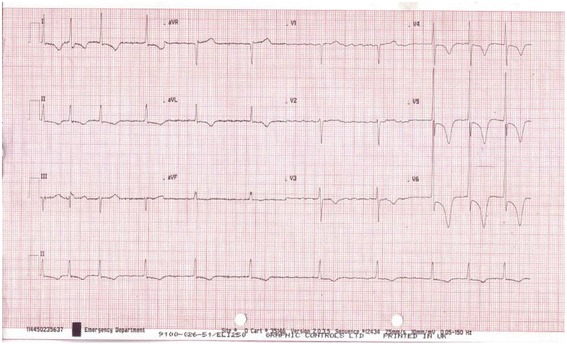
Patient’s electrocardiogram after suffering a stroke showing marked left ventricular hypertrophy

This is an interesting case because an ECG performed in December 2016 prior to the patient suffering a stroke (Fig. 1) showed atrial fibrillation with a narrow QRS complex. Importantly, this ECG did not meet the criteria for the diagnosis of LVH, using the Sokolow-Lyon indices [2]. However, an ECG performed 10 days after she suffered a stroke showed marked LVH (Fig. 2) using the same criteria.

We believe that ECG diagnosis of LVH (as it is the case with most patients) led to the diagnosis of HCM. However, what is surprising is the development of LVH a few days after suffering a stroke. To this effect, we decided to write this case report because it is highly unlikely that our patient developed LVH within 10 days, yet it was previously not evident on her prior ECG. However, it is plausible that suffering a stroke may have played a role in the ECG presentation of LVH. Later investigations confirmed the diagnosis of HCM in this patient.

## Case presentation

An 83-year-old white British woman presented to the emergency department with a 2-day history of exertional dyspnea and chest tightness. Her past medical history included permanent atrial fibrillation, hypertension, stroke, osteoarthritis, and a previous left shoulder operation. She has no family history of sudden death or cardiovascular disease. On examination, she had bibasal crepitations with a systolic a murmur loudest at the apex.

In-patient investigations include an ECG, blood tests, and a chest X-ray. She also underwent an echocardiogram, contrast echocardiogram, coronary angiogram, and cardiovascular MRI scan in addition.

History revealed that she had been previously admitted 10 days earlier with headache, vomiting, slurred speech, and left-sided facial droop. She was also found to be dyspneic, hypertensive, hypoxic with scattered wheeze, and with bibasal crepitations at the time. Her notes revealed that a bedside echocardiogram performed during that admission showed poor ejection fraction (< 20%). Furthermore, it was clear from her notes that she was treated with intravenous diuretics and glyceryl trinitrate infusion for acute pulmonary edema. A magnetic resonance imaging (MRI) scan of her head performed during the same admission confirmed acute left cerebellum infarct. The patient was subsequently treated for a cerebellar ischaemic stroke with loading dose of aspirin (300 mg), and 75 mg oral aspirin after 2 weeks. An ECG at the time showed no LVH. Her previous echocardiogram in 2015 showed mildly reduced left ventricular (LV) systolic function, thickened aortic valve, moderate tricuspid regurgitation, mild to moderate mitral regurgitation, and mild pulmonary regurgitation with dilated atria. A cardiovascular MRI scan in February 2017 at a regional tertiary center, showed severely impaired biventricular function and evidence of biatrial dilatation, plus a moderate to severe atrial scar.

### Diagnostic focus and assessment

An ECG performed during this admission showed atrial fibrillation, with inferolateral T wave inversion (new since February 2017), and LVH (new) (Fig. 2).

Blood results showed elevated troponin I of 95, 89 (normal < 30 ng/L) on repeat, and elevated brain natriuretic peptide (BNP) of 1175 (normal < 99 pg/mL).

**Fig. 3:**
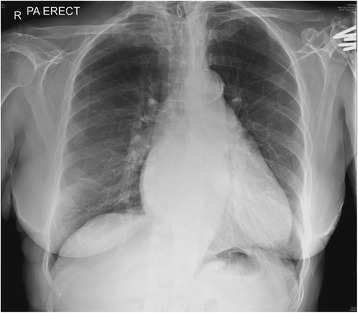
Patient’s chest X-ray on admission showing features consistent with interstitial edema

A chest X-ray (Fig. 3) showed prominent upper lobe pulmonary vessels in keeping with pulmonary vascular diversion, with interstitial thickening noted along the right base in keeping with septal edema.

**Fig. 4:**
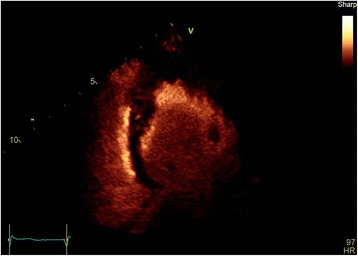
Apical four-chamber contrast echocardiography showing apical left ventricular hypertrophy

**Fig. 5:**
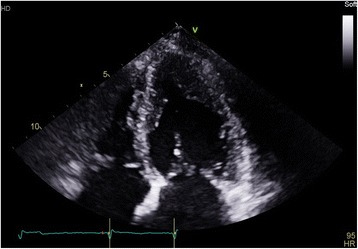
Apical four-chamber echocardiography showing apical left ventricular hypertrophy

**Fig. 6:**
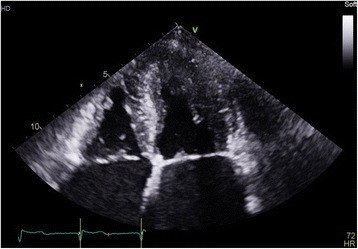
A different view of apical four-chamber echocardiography showing apical left ventricular hypertrophy

An echocardiogram and contrast echocardiogram (Figs. 4, 5 and 6) showed that her left ventricular systolic function visually appeared normal and biplane ejection fraction (EF) of 59% without contrast and 61% with contrast. Importantly, it showed marked hypertrophy of the mid to apical LV, appearance consistent with apical hypertrophic cardiomyopathy. The interventricular (IV) septum measured 1.3 cm (normal 0.6–1.2 cm), and the posterior wall measured 1.0 cm (normal 0.6–1.2 cm).

**Fig. 7:**
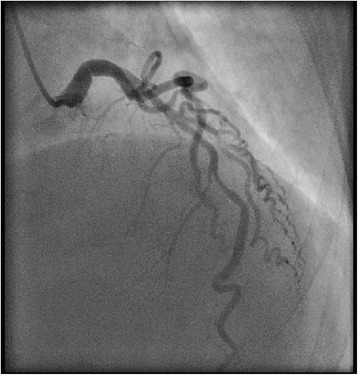
A coronary angiography showing the left main stem has eccentric shelf-type plaque with non-flow-limiting stenosis

Coronary angiography showed (Fig. 7) that the left coronary artery main stem has eccentric shelf-type plaque with non-flow-limiting stenosis. This was discussed with a tertiary center interventional cardiologist, and it was deemed nonsignificant to cause ischemia.

**Fig. 8:**
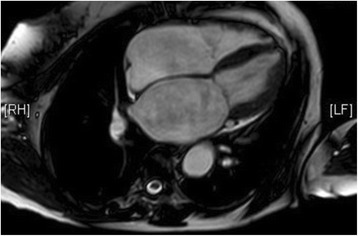
Cardiovascular magnetic resonance imaging showing apical left ventricular hypertrophy

**Fig. 9:**
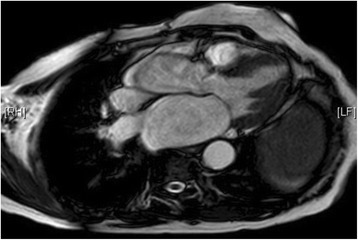
Cardiovascular magnetic resonance imaging showing apical left ventricular hypertrophy

Cardiovascular MRI (Figs. 8 and 9) reported findings highly suggestive of apical hypertrophic cardiomyopathy. The LV apex measurement was 12.4 cm. Our patient declined a stress perfusion scan to assess ischemic burden.

### Follow-up and outcomes

She was discharged on rivaroxaban, bisoprolol, and atorvastatin. She was also referred to the cardiomyopathy outpatient clinic. Family screening was recommended.

### Timeline

**Table:** 

December 2016	Presentation to a local hospital with shortness of breath
March 2017	Presents to a different hospital with headache, vomiting, and slurred speech
April 2017	Presents to a local district hospital with chest tightness and exertional dyspnea

## Discussion

We believe that ECG diagnosis of LVH was the first step in diagnosing apical HCM in this patient. It is well known that LVH is not progressive [1]. Therefore it is implausible that this patient developed LVH within 4 months. The factors responsible for the difference in pre- and post-stroke ECGs (Figs. 1 and 2) remain somewhat unclear.

We are convinced that LVH diagnosed on the patient’s post-stroke ECG was due to HCM rather than hypertension. This is because she has had hypertension for 20 years, and the pre-stroke ECG did not meet the criteria for LVH (Figs. 1 and 2).

Hypertrophic cardiomyopathy is a genetic heart muscle disease mainly (in about 70%) caused by mutations of the several cardiac sarcomere genes that encode vital proteins central to the contractile machinery of the heart [1]. It is characterized by LVH of various morphologies with an array of clinical presentations depending upon the location, extent, and distribution of the hypertrophy [1]. Recently, 11 genes with over 1500 mutations encoding the thick or thin myofilament proteins of the sarcomere and associated Z-disc have been identified [1, 3]. In addition, these varied mutations lead to heterogeneous phenotypic expression of HCM with respect to the aforementioned LVH morphologies as well as diverse clinical course that include sudden death, heart failure, and stroke [4, 5]. This clinical course is evident from the case presentation because our patient has had heart failure and stroke.

Its prevalence in a variety of ethnic populations around the world has previously been estimated to be 0.2% (1 out of every 500 adults) [6, 7]. However, subsequent studies using data from echocardiography, genetic testing, and cardiovascular MRI has estimated the prevalence to be closer to 0.5% (1 out of every 200 adults) [8].

Interestingly, the main abnormalities present in HCM include LV outflow obstruction, diastolic dysfunction, myocardial ischemia, and mitral regurgitation [1]. These structural and functional abnormalities produce a variety of symptoms for patients including chest pain, exertional dyspnea, fatigue, presyncope/syncope, and palpitations. Importantly, these symptoms are broadly categorized into those related to heart failure (90% of the cases), chest pain (25–30% of the cases), or arrhythmias (including supraventricular primarily atrial fibrillation, and ventricular arrhythmias) [1].

As was the case with this patient, atrial fibrillation was permanent, and her presenting symptoms are well recognized in HCM.

Electrocardiography, echocardiogram, and cardiovascular MRI form the basis of diagnosing HCM. In 90% of patients with HCM, ECG is abnormal although there is no specific diagnostic pattern. Main ECG findings include repolarization abnormalities, prominent Q waves, P wave abnormalities, left axis deviation, and deeply inverted T waves [1]. Findings from echocardiogram suggestive of HCM include asymmetrical LVH involving the septum and anterolateral wall, an increased LVOT gradient, and systolic anterior motion of the mitral valve leaflets associated with an increased LVOT gradient [1].

## Conclusions

An ECG performed 10 days after our patient suffered a stroke showed marked LVH which was not present 4 months prior to suffering a stroke. It is unlikely that LVH developed within 4 months because the time course of LVH is well documented in the literature. It is also not plausible that the LVH present was due to long-standing hypertension because it would have been present on the pre-stroke ECG.

Diagnosis of LVH was pivotal in diagnosing HCM. There is no clear explanation for the accelerated development of LVH within 4 months, particularly 10 days post-stroke. In addition, stroke may or may not have contributed to the LVH evident in the post-stroke ECG. This phenomenon has not been previously reported. The take-home messages from this case report are as follows: differential diagnosis of hypertrophic cardiomyopathy should be considered in people with ECG criteria for LVH. Cardiovascular MRI is an important diagnostic tool in identifying causes of left ventricular hypertrophy. Family screening should be recommended in patients with new diagnosis of hypertrophic cardiomyopathy.
